# Parasite fauna of Antarctic *Macrourus whitsoni* (Gadiformes: Macrouridae) in comparison with closely related macrourids

**DOI:** 10.1186/s13071-016-1688-x

**Published:** 2016-07-20

**Authors:** Julian Münster, Judith Kochmann, Sven Klimpel, Regina Klapper, Thomas Kuhn

**Affiliations:** Goethe-University (GU), Institute for Ecology, Evolution and Diversity, Senckenberg Biodiversity and Climate Research Centre (BiK-F), Senckenberg Gesellschaft für Naturforschung (SGN), Max-von-Laue-Str. 13, D-60438 Frankfurt/Main, Germany

**Keywords:** Antarctica, Feeding behaviour, Macrourinae, *Macrourus whitsoni*, Grenadier fish, Parasites, Digenea, Nematoda

## Abstract

**Background:**

The extreme, isolated environment within the Antarctic Convergence has fuelled the evolution of a highly endemic fauna with unique adaptations. One species known from this area is the Whitson’s grenadier *Macrourus whitsoni* (Regan, 1913). While closely related species occurring in the Northern Hemisphere were targets of a variety of studies, knowledge on *M. whitsoni* is scarce, including not only its ecology but also its parasite fauna. Parasites, an often overlooked but important component of every ecosystem, can provide important insights into host ecology, including feeding habits, food web interactions and distribution patterns. The aim of our study was to increase the currently limited knowledge on the ecology of *M. whitsoni* and its parasite life-cycles.

**Methods:**

In this study, parasite fauna and stomach content of 50 specimens of *M. whitsoni* were sampled off Elephant and King George Islands. Fish samples were morphological, food ecological and parasitological examined and parasites morphological and partly molecular identified. To evaluate the findings, results were compared with other macrourid species.

**Results:**

The parasite fauna of *M. whitsoni* revealed 9 genera and 17 species. Stomach content analysis indicated Amphipoda and Mysida as the primary food source. Considering the parasites of *M. whitsoni*, the highest diversity was found within the Digenea, while prevalence was highest for the Acanthocephala and Nematoda. The diverse parasite fauna of *M. whitsoni* together with the stomach content analysis, suggests a benthopelagic mode of life. Furthermore, an extensive evaluation of the parasite fauna of species of the Macrourinae was conducted, which is probably the most thorough one yet, to compare the findings with closely related host fish species. A similarity analysis revealed a strong connection between the parasite fauna composition and geographical distribution, with a clear separation between the parasite faunas in fishes sampled in the Pacific and the Atlantic Oceans.

**Conclusions:**

Due to the isolated habitat within the Antarctic Conversion, the parasite fauna of *M. whitsoni* differs clearly from those of closely related and closely occurring species of the genus *Macrourus*. Our study revealed an endemically dominated parasite fauna, with parasites often host-specific to *M. whitsoni.* The comparison with the faunas of other species of the Macrourinae revealed a largely endemic parasite fauna, which emphasizes again the isolated character of the Antarctic shelf regions.

**Electronic supplementary material:**

The online version of this article (doi:10.1186/s13071-016-1688-x) contains supplementary material, which is available to authorized users.

## Background

The Macrouridae is, with over 300 species, the dominant benthopelagic deep-sea fish family in terms of species as well as biomass [1–3]. Macrourids occur primarily at the continental slopes in depths between 200 and 2000 m. Some species can also be found in the abyssal plains and only a few inhabit the meso- and bathypelagic zones of the oceans. Macrourids are absent in high Arctic waters [1, 3, 4]. The feeding ecology of macrourids is highly diverse, depending on species, size, depth and the nature of the seabed [2]. Most species feed near the bottom, where they search for prey in the sediments, or hunt benthic Crustacea; only few prey on fish, cephalopods and euphausiids in the water column [2].

The family Macrouridae consists of four subfamilies: Bathygadinae, Macrouroidinae, Macrourinae and Trachyrincinae. While the species richness for three subfamilies is relatively low, the Macrourinae contains 28 genera and over 270 species, including the commercially exploited genera *Coryphaenoides* and *Macrourus* [2, 5]. Within the genus *Coryphaenoides*, species exhibit a cosmopolitan distribution, except in Antarctic waters. Within the genus *Macrourus* four out of five species (*M. caml*, *M. carinatus*, *M. holotrachys*, *M. whitsoni*) occur only in the southern hemisphere and one, *M. berglax*, in the North Atlantic [3, 5, 6]. While species of the North Atlantic have been the targets of a variety of studies (e.g. *Coryphaenoides rupestris* or *Macrourus berglax*), other macrourids are not as well studied. This is especially true for the Southern Ocean, where species such as *Macrourus whitsoni* and *M. caml* commonly occur within the Antarctic Convergence (except for Falkland Islands) [5].

Studies of fishes often focus on feeding habits. Combined, with their parasite fauna (e.g. parasite diversity and infection rates), these studies allow a better understanding of the host’s ecology and can help to elucidate the roles of different groups within food webs [7, 8]. For instance, metazoan parasites, especially helminths, have evolved complex life-cycles, including several hosts among different trophic levels, and are therefore deeply embedded within food webs [8]. Encircled by the Antarctic Convergence causing geographical and seasonal isolation, the Antarctic is a unique ecosystem, home to a multitude of endemic species that are forming a relatively simple food web consisting of phytoplanktonic primary producers, zooplanktonic primary consumers and a series of predators (e.g. fish, whales, seals, seabirds, detritivores) [9]. Here, parasite fauna and infection patterns of the Whitson’s grenadier, *M. whitsoni*, were assessed and combined with stomach content analysis in an effort to increase our currently limited knowledge of the ecology and parasite life-cycles relevant to this host. Data were then used to compare the parasite fauna of *M. whitsoni* with other closely related species within the genera *Coryphaenoides* and *Macrourus*; this should help produce a more comprehensive picture of the parasite fauna and the global role of the genera *Macrourus* and *Coryphaenoides* in oceanic food webs.

## Methods

### Sample collection

*Macrourus whitsoni* were sampled in March and April 2012 during the research cruise ANT XXVIII/4 on board of the German *RV Polarstern* in waters off King George and Elephant Island, Antarctica. Sampling was conducted with a bottom trawl at depths of 420.1 to 479.1 m with a towing time of 30 min and a speed of 2.8–4.1 kn (nautical miles/ hour). A total of 50 specimens of *M. whitsoni* were caught and stored at -40 °C immediately after capture for subsequent examination at the Institute of Ecology, Evolution and Diversity, Goethe University, Frankfurt, Germany. Prior to examination, each specimen was thawed and taxonomically identified using Gon & Heemstra [10].

### Morphological and parasitological examination

Total length (TL), preanal length (PAL), total weight (TW) and carcass weight (CW) were measured to the nearest 0.1 cm or 0.1 g, respectively. First, the eyes, fins, skin, gills as well as the nasal, buccal and branchial cavities were inspected for ectoparasites. Afterwards, the body cavity was opened and the internal organs, i.e. the liver, stomach, pyloric caeca, intestine and gonads, were dissected and checked for endoparasites using a stereomicroscope (Olympus SZ 61, at magnifications of 6.7–45). Stomach contents were removed for content analyses (see below). Parasites were isolated and host tissue was removed. Digenean, monogenean, cestode and acanthocephalan parasites were fixed in 4 % borax buffered formalin, preserved in 70 % ethanol (with 4 % glycerol) and morphologically identified using the existing keys and original descriptions [11–16]. Nematode specimens were directly preserved in absolute ethanol for subsequent molecular identification (see Additional file 1).

### Stomach content analyses

Food items were separated and identified to the lowest possible taxonomic level and grouped into taxonomic categories (e.g. subphylum). The dry weight of full and empty stomachs as well as the dry weight of the different food items and groups were recorded to the nearest 0.001 g. Frequency of occurrence (F in %), numerical percentage of prey (N in %) and the weight percentage of prey (W in %) were calculated following Hyslop [17]. The index of relative importance (IRI) was calculated based on these data [18].

### Data analyses

Statistical analyses were performed using the software Graphpad Prism v5.01. Parasitological and ecological terminology follow Bush et al. [19]: prevalence (P) defined as the relative number of fish infected with a specific parasite; intensity (I) as the number of individuals of a particular parasite species in a single infected host (given as a range); and mean intensity (MI) as the average intensity of a particular parasite species among the infected specimens of a particular host species.

### Metadata analysis

The data on the metazoan parasite fauna previously reported from the different macrourid species was collected by means of a search in Google Scholar and cross-checked with the references in the Web of Knowledge. For the search, the names of all known species of *Macrourus* and *Coryphaenoides*, along with the keywords “parasite”, “Digenea”, “Monogenea”, “Cestoda”, “Nematoda”, “Acanthocephala” and “Crustacea”, were used. Species names were checked using the World Register of Marine Species (www.marinespecies.org). Only unambiguous records were included. In addition to original publications, Klimpel et al. [20] was also utilized. Parasite species richness was calculated for each fish species and its correlation with the number of publications assessed. For a comparison of the parasite faunas in the different fish species, Bray-Curtis similarities were calculated using presence/absence data for the parasite species; based on these data, hierarchical cluster analysis was performed with the Primer 6 software [21].

Tables list species identified to the species level. Numbers of taxa are given as well, i.e. reflecting identification to order, class, family, genera or species level. However, these taxa counts are most likely an overestimation as unidentified specimens from different studies may have been counted as different taxa although they could be the same. Thus, only parasite unambiguously identified to the species level were used for Bray-Curtis calculations.

## Results

### Host biometric data and parasite infection data

The mean TL and PAL of the 50 examined specimens of *Macrourus whitsoni* was 23.14 cm (± standard deviation, SD, 5.0 cm), normality test: *P* = 0.74) and 7.7 (± 1.7 cm SD), respectively; the mean TW was 67.2 g (± 34.9 g SD) and CW = 50.9 g (± 27.7 g SD). Of the 50 examined fish, 43 (P = 84.0 %) were infected with a total of seven genera and ten species of parasite, consisting of 219 individual specimens (Table 1). Digeneans infecting the gastro-intestinal tract, had the highest diversity, with a total of 11 species. *Macruricotyle claviceps* was the only identified species of monogenean parasitizing the gills of *M. whitsoni.* Larval specimens of the acanthocephalan *Corynosoma bullosum* were found in the visceral cavity. The nematode *Pseudoterranova decipiens* E [22] (GenBank: KX378173, KX378174; reference accession number: KF017610.1 [23]), identified by molecular analysis, constitutes a new host record for *M. whitsoni*.

**Table 1:** Parasite fauna of *Macrourus whitsoni*. Parasites of *M. whitsoni* (*n* = 43) sampled in Antarctica (off Elephant and King George Islands). Species marked as unidentified were clearly recognised as distinct species. Shown are the site in host, prevalence (P in %), mean intensity (MI) and range for intensity (I)

Parasite	Life-cycle stage	Site in host	P (%)	MI (I)
Monogenea	A	G	44.0	1.9 (1–4)
*Macruricotyle clavipes*	A	G	44.0	1.9 (1–4)
Unidentified monogenean	A	G	2.0	1.0 (1)
Digenea	A	In, P	28.0	2.2 (1–7)
*Paralepidapedon dubium*	A	P	2.0	4.0 (4)
*Paralepidapedon awii*	A	P, In	2.0	1.0 (1)
*Paralepidapedon antacrctica*	A	P	2.0	1.0 (1)
*Lepidapedon brayi*	A	P	4.0	1.0 (1)
*Lepidapedon ninae*	A	P	2.0	1.0 (1)
*Gonocerca phycidis*	A	St	2.0	1.0 (1)
Unidentified digenean^a^	A	In, P, St	52.0	1.6 (1–7)
Nematoda	L	L, St	52.0	1.6 (1–6)
*Pseudoterranova decipiens* E	L		14.0	1.1 (1–2)
Unidentified nematode	L	L, St	42.0	1.7 (1–5)
Cestoda	A	I	2.0	1.0 (1)
*Parabothriocephalus johnstoni*	A	I	2.0	1.0 (1)
Acanthocephala	L	Bc	64.0	3.2 (1–13)
*Corynosoma bullosum*	L	Bc	64.0	3.2 (1–13)

*Abbreviations*: *A* adult, *L* larva, *G* gills, *In* intestine, *L* liver, *P* pyloric caeca, *St* stomach

^a^Presumably five different species

### Stomach content analyses

The stomach content analyses revealed that 92.3 % of the stomachs contained food items belonging to the Crustacea. Overall three food items, i.e. belonging to the Amphipoda, Isopoda and Mysida, were identified. The most frequent preys were amphipods (F = 30.8 %, IRI = 628.32) followed by mysids (F = 12.8 %, IRI = 256). Isopods were less frequent (Table 2). Due to the advanced state of digestion of most food items, identification to a lower taxonomic level was often not possible.

**Table 2:** Stomach content of *Macrourus whitsoni*. Results of stomach content analysis of 47 specimens of *M. whitsoni* sampled off Elephant and King George Islands, Antarctica. Frequency of occurrence (F in %), numerical percentage (N in %), weight percentage (W in %) and the index of relative importance (IRI) of the major prey groups are shown

Prey	F (%)	N (%)	W (%)	IRI
Crustacea	92.3	98.3	89.3	17,315.48
Amphipoda	30.8	18.0	2.4	628.32
Mysida	12.8	10.2	9.8	256.00
Isopoda	5.1	0.7	0.3	4.98
Unidentified	84.6	69.5	86.9	13,231.44
Unidentified	12.8	1.7	0.8	31.50

### Comparison of parasites reported from *Macrourus* spp. and *Coryphaenoides* spp. 

**Fig. 1:**
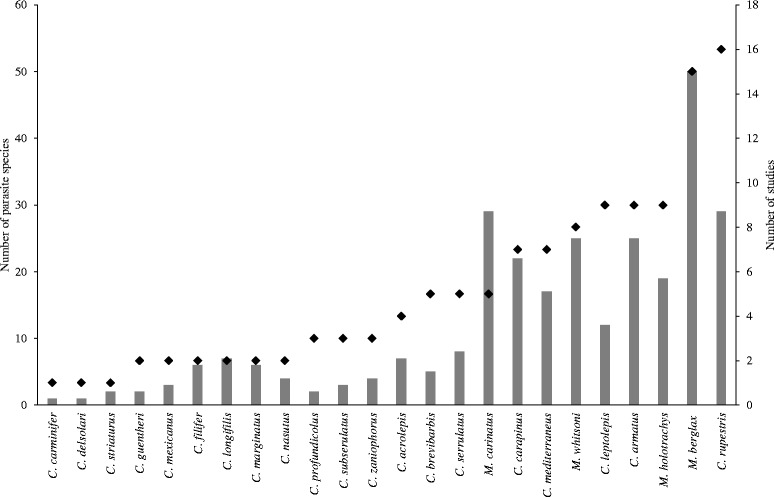
Parasite fauna and sampling effort. Number of recorded parasite species (*grey bars*, left y-axis) and number of publications on the parasite fauna of the specific fish species (*black rhombs*, right y-axis)

The search on Google Scholar resulted in 72 publications. In the search, only macrourid species with an already documented parasite fauna were included. The numbers of parasite taxa and publications were highly correlated (Spearman's rank correlation *r* = 0.862, *P* < 0.001) (Fig. 1). A total of 169 different metazoan parasite taxa were found among the 24 fish species included in the search, with 82 (48.5 %) parasite taxa being recorded only from one host species (Table 3). A total of 97 taxa (66 identified plus 31 unidentified species) were found reported for the four *Macrourus* spp. The greatest parasite diversity was found in *M. berglax* with 50 taxa (34 species) and in *M. carinatus* with 29 taxa (22 spp.) (Table 3, Additional file 2: Table S1). Within the genus *Coryphaenoides* containing 19 species, a total of 101 different parasite taxa (68 spp.) were recorded. *Coryphaenoides rupestris* and *C. armatus*, both Atlantic species, had the most diverse parasite faunas, with 30 and 25 different taxa, respectively; whereas parasite numbers for species of *Coryphaenoides* inhabiting the Pacific ranged between 3–8 species. Overall, the most diverse parasite groups were the Digenea with 78 taxa (57 spp.) followed by the Nematoda with 27 taxa (16 spp.). The Acanthocephala had the lowest diversity with only 7 taxa (5 spp.). Highest numbers of fish hosts were recorded for parasites known to have low host specificity (generalist parasites), e.g. *Gonocerca phycidis* occurring in 12 different fish species, *Glomericirrus macrouri* in nine, and *Gonocerca haedrichi* in seven fish species. Detailed information on the parasite taxa found only in one species or shared among different fish species can be found in Additional file 2: Table S1 [6, 13, 14, 20, 23–97]).

**Table 3:** Species richness of the major parasite groups in *Coryphaenoides* spp. and *Macrourus* spp. Data represent the total number and the number of distinct taxa (Unique) based on the review of the literature

Species	Mono	Dige	Cest	Nema	Acan	Crus	Total	Unique
*C. acrolepis*	1	5	0	1	0	0	7	2
*C. armatus*	1	12	2	6	0	4	25	7
*C. brevibarbis*	1	1	1	0	0	1	4	0
*C. carapinus*	1	11	2	5	1	2	22	4
*C. carminifer*	0	10	0	0	0	0	10	0
*C. delsolari*	0	0	0	0	0	1	1	1
*C. filifer*	0	5	0	0	0	1	6	1
*C. guentheri*	0	2	0	0	0	0	2	1
*C. leptolepis*	0	8	1	0	0	1	11	1
*C. longifilis*	0	2	0	0	0	0	7	3
*C. marginatus*	0	3	0	0	0	3	6	2
*C. mediterraneus*	0	8	2	8	0	1	19	4
*C. mexicanus*	0	2	0	0	0	0	2	0
*C. nasutus*	0	0	0	0	0	4	4	2
*C. profundicolus*	0	1	0	0	0	0	1	0
*C. rupestris*	1	14	5	6	1	3	30	4
*C. serrulatus*	4	5	0	0	0	1	10	3
*C. striaturus*	0	9	1	0	0	0	10	1
*C. subserrulatus*	2	0	0	0	0	1	3	1
*C. zaniophorus*	1	3	0	0	0	0	4	1
*M. berglax*	3	16	10	11	3	7	50	19
*M. carinatus*	2	10	5	8	2	2	29	7
*M. holotrachys*	4	8	0	0	1	6	19	5
*M. whitsoni*	2	10	4	4	2	3	25	13
different taxa	14	78	18	27	7	25	169	82

*Abbreviations*: *Mono* Monogenea, *Dige* Digenea, *Cest* Cestoda, *Nema* Nematoda, *Acan* Acanthocephala, *Crus* Crustacea

**Fig. 2:**
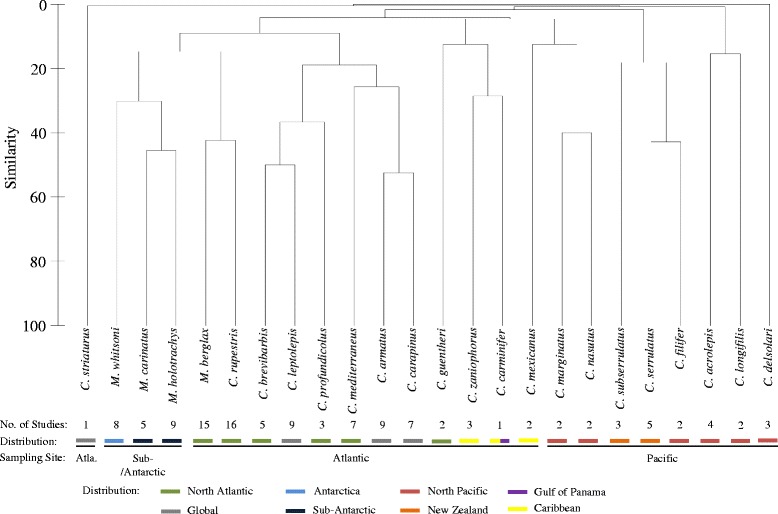
Parasite data based dendrogram for species of *Coryphaenoides* and *Macrourus*. Dendrogram for hierarchical clustering of *Macrourus whitsoni* sampled in this study and other *Macrourus* as well as *Coryphaenoides* species from the literature. Shown are Bray-Curtis similarities between fish species based on presence/absence data of parasite taxa as well as the number of studies on the different fish species and the geographical distribution of studies

The hierarchical cluster analysis based on Bray-Curtis similarity matrix revealed one cluster for species sampled in the Atlantic (plus two species from the North Pacific) (Fig. 2). This main cluster is subdivided into three subclusters. Thus, seven species of *Coryphaenoides* clustered together with *Macrourus* spp*.* In the Atlantic cluster within the genus *Macrourus*, the species *M. carinatus* and *M. holotrachys* exhibited the greatest similarity (45.4 %). These were followed by *M. holotrachys* and *M. whitsoni* (similarity of 35.9 % ) and *M. carinatus* and *M. whitsoni* (similarity of 24.5 %). Overall, *M. berglax* shared the lowest similarities with the three other *Macrourus* species (19.1 % with *M. carinatus*, 8.8 % with *M. whitsoni* and 6.3 % with *M. holotrachys*, respectively), but revealed the greatest similarity with *C. rupestris* (genus *Coryphaenoides*) (42.2 %). Among the species of *Coryphaenoides*, the parasite faunas in *C. armatus* and *C. carapinus* were identified as being most similar (similarity of 52.4 %). Apart from the main cluster, two smaller clusters of *Coryphaenoides* spp. from Pacific waters were found. Here, *C. filifer* and *C. serrulatus* had the highest similarity (42.8 %). *Coryphaenoides acrolepis* showed the most pronounced similarity patterns with *C. longifilis* (15.4 %) with which it formed a cluster, whereas *Coryphaenoides serrulatus* and *C. subserrulatus*, occurring in the same waters had a similarity of 36.4 % in their parasite fauna. *Coryphaenoides delsolari* and *C. striaturus*, did not fit to either of the clusters and showed no similarity with the other species.

## Discussion

As suggested by Polyanskii [98], parasites in higher, Nordic latitudes are often generalists, an assumption that was later on extended upon parasites from Antarctic waters [99]. However, most parasites in the Antarctic and sub-Antarctic regions are endemic and for many of them, fish serve as final hosts [100]. The aim of this study was to investigate the parasite fauna and diet of *Macrourus whitsoni* from the Antarctic region and to compare it with closely related species within the subfamily Macrourinae. Sixteen different metazoan parasite species were found in the 50 specimens of *M. whitsoni* examined. Although a total number of 25 metazoan parasites are known so far from *M. whitsoni* (Additional file 2: Table S1) mainly from studies in the Weddell Sea and off King George Island [49], the numbers found here still suggest that *M. whitsoni* is one of the most diversely parasitized deep-sea fish species. In comparison, only six parasite species are known for *Gymnodraco acuticeps* (Bathydraconidae) occurring in the same region [101–103]. The parasite fauna of *M. whitsoni* found in our study consists of taxa with high host specificity and a restricted distribution and is, to our current knowledge, host-specific either for *Macrourus* spp. or only *M. whitsoni*. However, some generalist parasites with cosmopolitan distribution were also found. Our literature data search revealed that macrourid species, at least those that are well-studied, have diverse parasite faunas, that might be ascribed to a high biomass of benthic organisms and therefore a high number of potential intermediate hosts in the deep-sea habitats [20, 26].

The parasite fauna of *M. whitsoni* consists to a large extent of species only known from Antarctic waters, with some of these parasite species using *M. whitsoni* as their only definitive host [13, 49, 50]. Most of these endemic species are digeneans, also the most diverse group found in this study, with all six identified species maturing in *M. whitsoni*. Digeneans are considered as the most species-rich parasite group in the waters off King George and Elephant Islands, with most of them maturing in teleosts ([49, 50, 100], this study). Forty-five species of digenean are known from these waters, belonging to the superfamilies Hemiuroidea and Allocreadioidea [50, 100]. Of these, 30 appear to be endemic to Antarctic waters while four are known to be cosmopolitan or bipolar [104]. Only *Gonocerca phycidis* has a cosmopolitan distribution with a broad host and depth ranges [20, 49], whereas the other species found in our study (*Lepidapedon brayi*, *L. ninae*, *Paralepidapedon awii*, *P. antarctica* and *P. dubium*) are only known from *M. whitsoni* so far [13, 100]. Thus, they might be endemic to Antarctic waters with distinct host specificity for *M. whitsoni* as suggested previously [49]. Some digeneans, presumably five species, could not be identified, due to their poor condition. Only one nematode, *Pseudoterranova decipiens* E [22], could be identified. *Pseudoterranova decipiens* E can be characterized as a species with low intermediate host specificity in Antarctic waters; its distribution and abundance generally depend on seal populations within the area [105, 106]. Although this parasite is widely distributed in Antarctic waters and known to occur in other fish intermediate hosts in these waters [107], it has not been found in *M. whitsoni* before [13, 49, 106]. Thus, it should be considered a new host record. The presence of this parasite indicates demersal feeding behaviour of *M. whitsoni*, as, contrary to other nematode larvae such as *Anisakis* spp. or *Contracaecum radiatum*, larvae of *P. decipiens* are unable to swim and therefore sink to the ground to follow a benthic life-cycle [106, 108]. Within the Acanthocephala, *Corynosoma bullosum* was the only recorded species in our samples of *M. whitsoni*. This is not surprising as *C. bullosum* is one of the few species that are typically found on the open-sea shelf, while most acanthocephalans in Antarctic waters are abundant in inshore regions [12] and likely to be absent from deep sea-fish like *M. whitsoni*. Generally, *C. bullosum* has a circumpolar distribution in the Antarctic and possesses, together with other members of its genus, a relatively complex life-cycle, including Amphipoda, e.g. *Bovallia gigantean* and *Waldeckia obesa* as first intermediate host [109, 110]. Apart from *M. whitsoni*, other fish species, e.g. *Chaenocephalus aceratus* or *Dissostichus eleginoides*, can serve as paratenic hosts for this parasite, whereas seals, e.g. *Mirounga leonine* or *Leptonychotes weddellii* serve as definitive hosts [12, 111–113]. Confirming earlier findings from the Weddell Sea and off King George Island by Walter et al. [49], the only species of monogenean found in the samples was *Macruricotyle clavipes*. The Monogenea, as known so far, parasitize only the Macrouridae, mostly *Macrourus* spp. (*M. clavipes*, *M. holotrachys*) in Antarctic and sub-Antarctic waters [49]. Among the cestodes, one adult specimen of *Parabothriocephalus johnstoni* was found in one individual of *M. whitsoni*. This cestode species seems to be endemic to Antarctic waters and is so far only known from *M. whitsoni* [49, 101].

Trophic studies on *M. whitsoni* are scarce, but it seems that Amphipoda and Euphausiacea are most likely the main preys throughout its life history ([106]; this study). Teleosts, however, were not of importance, although they are reported in the diet of different *Macrourus* spp. from the Ross Sea slope [114]. One explanation might be an ontogenetic shift in diet when reaching a specific size, thus, fish as prey items might only occur in specimens over TL of 30 cm, which was the maximum length sampled here. These dietary patterns are in accordance with other trophic studies from Antarctic waters where amphipods were recognized as the main food source for many fish species [115]. Crustaceans are important intermediate hosts, especially for nematodes and acanthocephalans, but usually show low infection rates (e.g. *Corynosoma bullosum* showed a prevalence of 0.49 % in the amphipod *Bovallia gigantea* and 0.08 % in *Waldeckia obesa* in the Admiralty Bay and *Corynosoma pseudohamanni* was found with a prevalence of 0.56 % in the amphipod *Cheirimedon femonratus* off South Shetland Islands [109, 116, 117]. Therefore, fish preying on crustaceans, such as *M. whitsoni*, are often only lightly infected (Nematoda: P = 52.0 %, MI = 1.65; Acanthocephala: P = 64.0 %, MI = 3.22), while piscivorous fish such as *Dissostichus eleginoides*, an important predator of *M. whitsoni* [118], can be heavily infected [111].

Using presence/absence data for parasite fauna composition and the Bray-Curtis similarity index, macrourid species clustered in one main cluster with species from the Atlantic (and two from the Pacific) and two smaller clusters, including species solely from Pacific waters. This pattern may be explained by the fact that species occurring closely or in the same waters would share more parasite species than species living further apart or in different ocean basins. Considering species in the Atlantic cluster, three subclusters were distinguished. One of these three clusters consisted of one species from the North Atlantic (*Coryphaenoides guentheri*), two species from Caribbean waters (*C. mexicanus* and *C. zaniophorus*), one species occurring in the Caribbean as well as Pacific waters off Middle America (*C. carminifer*) and two species from the Pacific (*C. marginatus* and *C. nasutus*). The clustering of species solely inhabiting the Pacific with those from the Atlantic might be affected by sampling bias (low sampling effort in the Pacific overall) and an increase of sampling might reveal closer similarities with other species from the Pacific. However, the two Pacific species (*C. marginatus* and *C. nasutus*) clustered together with species from the Caribbean and Panama waters and not with other Atlantic species, indicating that distance might play a role for similarity in parasite species. In the case of cosmopolitan fish species, e.g. *C. armatus*, parasite fauna showed greater similarities with those of fish species from the Atlantic than from the Pacific. However, this might only reflect a sampling bias as most of the studies on these species have taken place in the Atlantic (e.g. *C. armatus*: [57, 58]; *C. carapinus*: [57, 67]). Species from the Antarctic and sub-Antarctic regions showed greater similarities of their parasite faunas with species from Atlantic rather than Pacific waters. Between both *Macrourus* spp. inhabiting the sub-Antarctic and temperate waters around the Antarctic Convergence (*M. carinatus*: circumpolar; *M. holotrachys*: off the South American coast) [3, 5], the literature data revealed a parasite fauna with only 41.7 % similarity, whereas a more similar fauna in closely related species, occurring in the same marine geographic locality would be expected. One explanation for this dissimilarity might be their different feeding habits. *Macrourus carinatus* forages in the pelagic realm at depths shallower than 900 m while *M. holotrachys* feeds in the demersal zone at depths deeper than 1000 m [119]. Thus, their niches and potential parasite intermediate hosts do not overlap. Similar spatial partitioning can be expected in other regions where closely related macrourids occur (e.g. *M. caml* and *M. whitsoni*). Despite their close geographical distribution, the parasite fauna of *M. whitsoni* differs from that of *M. carinatus* and *M. holotrachys*. The reason might be found in the hydrographic characteristics of the Antarctic Convergence, which functions as a barrier for most animals, including fish, with *M. whitsoni* occurring within and *M. carinatus* and *M. holotrachys* outside of this barrier. While marine mammals (e.g. cetaceans, pinnipeds) can overcome this barrier and with them their parasites (e.g. different nematodes), for teleosts and their affiliated parasites, e.g. most digeneans, this is not possible. This explains the high percentage of animals being unique and endemic within this border [120]. Due to its isolated location geographically, oceanographically, bathymetrically and thermally, the Antarctic shelf is highly valuable for studying evolutionary mechanisms and can be compared to ancient rift lakes (e.g. Lake Tanganyika) with endemic species flocks [121]. To extend our knowledge and to test whether the same pronounced endemic patterns occur in other fish species inhabiting this unique ecosystem, further studies are underway.

## Conclusions

The study helped to shed light on the remote and isolated Antarctic realm, with a focus on the ecology of *Macrourus whitsoni* and its diverse parasite fauna. The comparison with closely related species of *Coryphaenoides* and *Macrourus* helped to emphasize the endemically dominated parasite fauna within the Antarctic Convergence. We hope that our findings may not only help generating knowledge on the isolation patterns of *Macrourus whitsoni*, but also stimulate further research on other deep-sea fish species in this unique environment.

## Additional files

Additional file 1: PCR amplification and species identification. (DOCX 16 kb) Additional file 2: Table S1. Parasite taxa in species of *Coryphaenoides* and *Macrourus* based on literature data. (DOCX 57 kb)
