# Supplementary material for: Parasite fauna of Antarctic Macrourus whitsoni (Gadiformes: Macrouridae) in comparison with closely related macrourids
Source: Parasit Vectors. 2016 Jul 20;9:403. doi: 10.1186/s13071-016-1688-x (PMC4955115; doi:10.1186/s13071-016-1688-x)
Supplement: Additional file 1: — PCR amplification and species identification. (DOCX 16 kb) [file 13071_2016_1688_MOESM1_ESM.docx]

**Additional file 1.** PCR amplification and species identification

Genomic DNA was isolated and purified from anisakid larvae by using a genomic DNA extraction kit (Peqlab Biotechnology GmbH, Germany) following the instructions of the manufacturer. For the molecular biological species identification a rDNA marker, including the ITS-1, 5.8S and ITS-2, was used. For the amplification of ITS primers TK1 (5'-GGC-AAA-AGT-CGT-AAC-AAG-GT-3') and NC2 (5'-TTA-GTT-TCT-TTT-CCT-CCG-CT-3') were used [1,2]. The PCR- reactions (50 µl) included 25 µl Master-Mix (Peqlab Biotechnology GmbH, Germany) containing dNTP, MgCl2, Buffer and Taq-Polymerase, 3 µl of each primer (10 pmol µl^-1^), 14 µl ddH_2_O and 5 µl genomic DNA. The PCR reaction were all performed in a thermocycler (Peqlab, Germany) under the following conditions*:* ITS-1/5.8S/ITS-2: an initial denaturation at 95°C for 120 sec, 40 cycles of 94°C for 20 sec (denaturation), 51°C for 20 sec (annealing), 72°C for 50 sec (extension), followed by a final extension at 72°C for 5 min. In each PCR run a samples without DNA were included. PCR products were examined on 1% agarose gels. To estimate the size of the PCR products a 100 bp ladder marker (peqGOLD, Germany) was used. PCR products were purified with Cycle-Pure Kit (Peqlab Biotechnology GmbH, Germany). Afterwards a total volume of 7 ml, including 2 µl primer (individually) and 5 µl of the PCR product (250 ng/µl) were sequenced by Seqlab Sequence Laboratories GmbH (Goettingen Germany). Sequences were compared with Genbank data using BLASTn [3].

1. Kuhn T, García-Màrquez J, Klimpel S. Adaptive radiation within marine anisakid nematodes: a zoogeographical modeling of cosmopolitan, zoonotic parasites. PLoS One. 2011;6:e28642.

2. Zhu X, D’Amelio S, Paggi L, Gasser RB. Assessing sequence variation in the internal transcribed spacers of ribosomal DNA within and among members of the Contracaecum osculatum complex (Nematoda: Ascaridoidea: Anisakidae). Parasitol. Res. 2000;86:677–683.

3. Altschul SF, Gish W, Miller W, Myers EW, Lipman DJ. Basic local alignment search tool. J. Mol. Biol. 1990;215:403–410.
