# Supplementary material for: Parasite fauna of Antarctic Macrourus whitsoni (Gadiformes: Macrouridae) in comparison with closely related macrourids
Source: Parasit Vectors. 2016 Jul 20;9:403. doi: 10.1186/s13071-016-1688-x (PMC4955115; doi:10.1186/s13071-016-1688-x)
Supplement: Additional file 2: Table S1. — Parasite taxa in species of Coryphaenoides and Macrourus based on literature data. (DOCX 57 kb) [file 13071_2016_1688_MOESM2_ESM.docx]

Additional file 2. Table S1. Parasite taxa in species of *Coryphaenoides* and *Macrourus* based on literature data. Parasite species or taxa that are only found in one fish species are marked by an asterisk (*).

| **Host** | **Parasite** | **Reference** |
| --- | --- | --- |
| ***Macrourus berglax*** | **Digenea (16)** |  |
| North Atlantic | *Bathycreadium flexicollis** | [1] |
|  | *Derogenes varicus* | [2–4] |
|  | *Dinosoma* sp. | [5] |
|  | *Genolinea laticauda** | [2–4,6] [4] |
|  | *Gibsonia borealis** | [3,4,7] |
|  | *Gonocerca crassa* | [2] |
|  | *Gonocerca kobayashii* | [5] |
|  | *Gonocerca phycidis* | [3,6] |
|  | *Gonocerca* sp. | [8] |
|  | *Hemiurus levinseni** | [2] |
|  | *Lecithophyllum botryophorum* | [2] |
|  | *Lepidapedon elongatum* | [2] |
|  | *Lepidapedon* sp. | [3–5] |
|  | *Steringophorus foliatus** | [5] |
|  | *Steringotrema pagelli** | [4] |
|  | Digenea indet. | [3,4] |
|  | **Monogenea (3)** |  |
|  | *Cyclocotyloides pinguis* | [2,5] |
|  | *Macrouridophora macruri* | [3,4] |
|  | *Macrouricotyle newfoundlandiae* | [9] |
|  | **Cestoda (10)** |  |
|  | *Diphyllobothrium* sp.* | [10] |
|  | *Eubothrium* sp.* | [10] |
|  | *Grillotia erinaceus* | [3,4] |
|  | *Hepatoxylon trichiuri** | [10] |
|  | *Nybelinia* sp. | [10] |
|  | *Parabothriocephalus macruri* | [3,4,9] |
|  | *Philobythos atlanticus* | [2] |
|  | Pseudophyllidea indet | [2] |
|  | *Scolex pleuronectis* | [2–5] |
|  | Trypanorhyncha indet. | [10] |
|  | **Nematoda (12)** |  |
|  | *Anisakis simplex* | [3] |
|  | *Anisakis* sp. | [2,4] |
|  | *Ascarophis* sp. | [3,4] |
|  | *Capillaria gracilis** | [2–4] |
|  | *Hysterothylacium aduncum* | [2–4] |
|  | *Hysterothylacium* sp.* | [8] |
|  | *Ichthyofilaria bergensis* | [4] |
|  | *Neoascarophis macrouri* | [3,4,11] |
|  | *Pseudoterranova decipiens* s.l.* | [3,4] |
|  | *Spinitectus oviflagellis* | [3,4,12] |
|  | *Spinitectus* sp.* | [8] |
|  | Nematoda indet. | [3,4] |
|  | **Acanthocephala (3)** |  |
|  | *Corynosoma wegeneri** | [4] |
|  | *Echinorhynchus gadi* | [1–3];  [4] |
|  | *Echinorhynchus* sp. | [8] |
|  | **Crustacea (7)** |  |
|  | *Chondracanthodes radiatus* | [3,4,13] |
|  | *Clavella adunca* | [2–4,13] |
|  | *Clavellomimus macruri** | [2] |
|  | *Lophoura bouvieri** | [4] |
|  | *Nectobrachia producta** | [4] |
|  | *Peniculus clavatus** | [4] |
|  | *Sphyrion* sp.* | [14,15] |
|  |  |  |
| ***Macrourus carinatus*** | **Digenea (10)** |  |
| Sub-Antarctic | *Brachyphallus crenatus* | [16] |
|  | *Elytrophalloides oatesi** | [16,17] |
|  | *Genolinea bowersi** | [16,17] |
|  | *Gibsonia hastata* | [16,17] |
|  | *Glomericirrus macrouri* | [16] |
|  | *Gonocerca haedrichi* | [16] |
|  | *Gonocerca phycidis* | [16] |
|  | *Lepidapedon lebouri* | [16] |
|  | *Lepidapedon taeniatum** | [16] |
|  | *Paralepidapedon lepidum* | [16,17] |
|  | **Monogenea (2)** |  |
|  | *Macrouridophora attenuata** | [18,19] |
|  | *Macruricotyle claviceps* | [16] |
|  | **Cestoda (5)** |  |
|  | *Grillotia erinaceus* | [16] |
|  | *Lacistorhynchus tenuis** | [16] |
|  | *Parabothriocephalus macruri* | [16] |
|  | *Phyllobothrium* sp.* | [16] |
|  | *Scolex pleuronectis* | [16] |
|  | **Nematoda (8)** |  |
|  | *Anisakis simplex* | [16] |
|  | *Ascarophis nototheniae* | [16] |
|  | *Capillaria* sp. | [16] |
|  | *Contracaecum* sp. | [16] |
|  | *Hysterothylacium aduncum* | [16] |
|  | *Neoascarophis sphaerocaudata* | [16] |
|  | *Neoascarophis* sp. | [16] |
|  | *Philometra* sp.* | [16] |
|  | **Acanthocephala (2)** |  |
|  | *Echinorhynchus longiproboscis* | [16] |
|  | Acanthocephala indet. | [16] |
|  | **Crustacea (2)** |  |
|  | *Chondracanthodes radiatus* | [16] |
|  | *Clavella adunca* | [16] |
|  |  |  |
| ***Macrourus holotrachys*** | **Digenea (8)** |  |
| Sub-Antarctic | *Brachyphallus crenatus* | [20] |
|  | *Gibsonia hastata* | [21] |
|  | *Glomericirrus macrouri* | [20] |
|  | *Gonocerca haedrichi* | [20] |
|  | *Gonocerca phycidis* | [21] |
|  | *Lecithochirium* sp.* | [21] |
|  | *Lepidapedon lebouri* | [20,22] |
|  | *Paralepidapedon lepidum* | [21] |
|  | **Monogenea (4)** |  |
|  | *Diclidophora* sp. | [23] |
|  | *Macruricotyle claviceps* | [24] |
|  | *Syncoelicotyloides macruri** | [25] |
|  | Monogenea indet.* | [26] |
|  | **Acanthocephala (1)** |  |
|  | *Echinorhynchus longiproboscis* | [27] |
|  | **Crustacea (6)** |  |
|  | *Chondracanthodes tuberofurcatus* | [26] |
|  | *Clavella adunca* | [26] |
|  | *Diocus semilunaris** | [26] |
|  | *Lophoura szidati* | [28] |
|  | *Sphyrion lumpi** | [26] |
|  | Crustacea indet. | [23] |
|  |  |  |
| ***Macrourus whitsoni*** | **Digenea (10)** |  |
| Antarctica | *Glomericirrus macrouri* | [29,30] |
|  | *Gonocerca haedrichi* | [30] |
|  | *Gonocerca phycidis* | [29,30] |
|  | *Lepidapedon brayi** | [29] |
|  | *Lepidapedon ninae** | [29] |
|  | *Lepidapedon* sp. | [30] |
|  | *Paralepidapedon antarcticum** | [31] |
|  | *Paralepidapedon awii** | [29,30] |
|  | *Paralepidapedon dubium** | [31] |
|  | *Postlepidapedon opisthobifurcatum** | [29,30] |
|  | **Monogenea (2)** |  |
|  | *Macruricotyle claviceps* | [30] |
|  | *Macruricotyle whitsonii** | [19] |
|  | **Cestoda (4)** |  |
|  | *Parabothriocephalus johnston*i* | [30,32] |
|  | Diphyllobothriidae indet.* | [30] |
|  | *Scolex pleuronectis* | [30] |
|  | Tetraphyllidea indet. | [33] |
|  | **Nematoda (4)** |  |
|  | *Capillaria* sp. | [30] |
|  | *Contracaecum osculatum* s.l.* | [30] |
|  | *Paranisakiopsis* cf. *australiensis* | [30] |
|  | *Pseudoterranova decipiens* E* | this study |
|  | Nematoda indet. | [30] |
|  | **Acanthocephala (2)** |  |
|  | *Corynosoma bullosum** | [30,34]; this study |
|  | *Echinorhynchus petrotschenkoi** | [34] |
|  | **Crustacea (3)** |  |
|  | *Chondracanthodes tuberofurcatus* | [30] |
|  | *Clavella adunca* | [30] |
|  | *Lophoura szidati* | [30] |
|  |  |  |
| ***Coryphaenoides acrolepis*** | **Digenea (5)** |  |
| North Pacific | *Dinosoma oregonense** | [35] |
|  | *Dinosoma pectorale** | [35] |
|  | *Lepidapedon abyssense* | [36] |
|  | *Lepidapedon luteum* | [35] |
|  | *Paraccacladium jamiesoni* | [36] |
|  | **Monogenea (1)** |  |
|  | *Cyclocotyloides pinguis* | [37] |
|  | **Nematoda (1)** |  |
|  | *Neoascarophis insulana* | [38] |
|  |  |  |
| ***Coryphaenoides armatus*** | **Digenea (12)** |  |
| global | *Genolinea* sp.* | [39] |
|  | *Glomericirrus macrouri* | [39,40] |
|  | *Gonocerca haedrichi* | [39,40] |
|  | *Gonocerca phycidis* | [39,40] |
|  | *Lepidapedon beveridgei* | [41,42] |
|  | *Lepidapedon discoveryi** | [42] |
|  | *Lepidapedon elongatum* | [39,43] |
|  | *Lepidapedon gaevskayae** | [41,42] |
|  | *Lepidapedon* sp. | [39] |
|  | *Neolepidapedon* sp. | [39] |
|  | *Profundivermis intercalarius** | [44] |
|  | Digenea indet. | [39] |
|  | **Monogenea (1)** |  |
|  | *Diclidophoropsis* sp.* | [39] |
|  | **Cestoda (2)** |  |
|  | *Grillotia rowei* | [39,40,45] |
|  | Tetraphyllidea indet. | [39] |
|  | **Nematoda (6)** |  |
|  | *Ascarophis* sp. | [39] |
|  | *Heterotyphlum* sp.* | [39] |
|  | *Hysterothylacium* sp. | [39] |
|  | *Neoascarophis* sp. | [39] |
|  | *Paranisakiopsis lintoni* | [39] |
|  | *Spinitectus oviflagellis* | [39] |
|  | **Crustacea (4)** |  |
|  | *Chondracanthodes deflexus* | [13,39,46] |
|  | *Lophoura pentaloba* | [13] |
|  | *Lophoura* sp.* | [39] |
|  | Crustacea indet. | [39] |
|  |  |  |
| ***Coryphaenoides brevibarbis*** | **Digenea (1)** |  |
| North Atlantic | *Steringophorus thulini* | [47] |
|  | **Monogenea (1)** |  |
|  | *Cyclocotyloides bergstadi* | [48] |
|  | **Cestoda (2)** |  |
|  | *Grillotia rowei* | [45] |
|  | **Crustacea (1)** |  |
|  | *Chondracanthodes deflexus* | [13] |
|  |  |  |
| ***Coryphaenoides carapinus*** | **Digenea (11)** |  |
| global | *Glomericirrus macrouri* | [39,40] |
|  | *Gonocerca haedrichi* | [39] |
|  | *Gonocerca phycidis* | [39] |
|  | *Lepidapedon luteum* | [39] |
|  | *Lepidapedon* sp. | [39] |
|  | *Neolepidapedon* sp. | [39] |
|  | *Plagioporus* sp. | [39] |
|  | *Podocotyle harrisae* | [49] |
|  | *Podocotyle schistotesticulata** | [49] |
|  | *Steringophorus furciger** | [47,50] |
|  | *Steringophorus* sp. | [39] |
|  | **Monogenea (1)** |  |
|  | *Cyclocotyloides* sp.* | [39] |
|  | **Cestoda (2)** |  |
|  | *Grillotia rowei* | [50,51] |
|  | Pseudophyllidea indet | [39] |
|  | **Nematoda (5)** |  |
|  | *Ascarophis* sp. | [39] |
|  | *Hysterothylacium reliquens** | [39] |
|  | *Neoascarophis* sp. | [39] |
|  | *Paranisakiopsis lintoni* | [39] |
|  | *Spinitectus oviflagellis* | [39] |
|  | **Acanthocephala (1)** |  |
|  | *Echinorhynchus* sp. | [39] |
|  | **Crustacea (2)** |  |
|  | *Chondracanthodes deflexus* | [13,39] |
|  | Crustacea indet. | [39] |
|  |  |  |
| ***Coryphaenoides carminifer*** | **Digenea (1)** |  |
| Caribbean, Gulf of Panama | *Lepidapedon mexicanense* | [52] |
|  |  |  |
| ***Coryphaenoides desolari*** | **Crustacea (1)** |  |
| East Pacific | *Lateracanthus quadripedis** | [53] |
|  |  |  |
| ***Coryphaenoides filifer*** | **Digenea (5)** |  |
| North Pacific | *Lepidapedon cascadense* | [36] |
|  | *Lepidapedon filiforme** | [36] |
|  | *Lepidapedon luteum* | [36] |
|  | *Lepidapedon oregonense* | [36] |
|  | *Lepidapedon yaquina* | [36] |
|  | **Crustacea (1)** |  |
|  | *Rebelula pentaloba* | [54] |
|  |  |  |
| ***Coryphaenoides guentheri*** | **Digenea (2)** |  |
| North Atlantic | *Lepidapedon sommervillae** | [42] |
|  | *Lepidapedon* sp. | [50] |
|  |  |  |
| ***Coryphaenoides leptolepis*** | **Digenea (6)** |  |
| global | *Glomericirrus macrouri* | [40] |
|  | *Lepidapedon luteum* | [36] |
|  | *Lepidapedon zubchenkoi** | [41,42,55] |
|  | *Lepidapedon* sp. | [42] |
|  | *Neolepidapedon* sp. | [39] |
|  | *Steringophorus thulini* | [47] |
|  | **Cestoda (1)** |  |
|  | *Grillotia rowei* | [51] |
|  | **Crustacea (1)** |  |
|  | *Chondracanthodes deflexus* | [13] |
|  |  |  |
| ***Coryphaenoides longifilis*** | **Digenea (7)** |  |
| North Pacific | *Derogenes macrostoma** | [56] |
|  | *Dinosoma lophiomi** | [56] |
|  | *Gonocerca crassa* | [56] |
|  | *Lepidapedon abyssense* | [36] |
|  | *Lepidapedon elongatum* | [56] |
|  | *Lepidophyllum* sp. | [56] |
|  | *Tellervotrema katadara** | [56] |
|  |  |  |
| ***Coryphaenoides marginatus*** | **Digenea (3)** |  |
| North Pacific | *Glomericirrus amadai** | [57] |
|  | *Gonocerca phycidis* | [57] |
|  | *Lepidapedon luteum* | [57] |
|  | **Crustacea (3)** |  |
|  | *Clavella longicauda** | [58] |
|  | *Clavella okamurai* | [58] |
|  | *Clavella sokodara* | [58] |
|  |  |  |
| ***Coryphaenoides mediterraneus*** | **Digenea (7)** |  |
| North Atlantic | *Allopodocotyle margolisi** | [59,60] |
|  | *Glomericirrus macrouri* | [60] |
|  | *Gonocerca minuta** | [60] |
|  | *Lepidapedon beveridgei* | [42,55,60] |
|  | *Steringophorus thulini* | [47,60] |
|  | *Steringotrema* sp.* | [60] |
|  | Digenea indet. | [60] |
|  | **Cestoda (1)** |  |
|  | *Scolex pleuronectis* | [60] |
|  | **Nematoda (8)** |  |
|  | *Anisakis simplex* | [60] |
|  | *Ascarophis nototheniae* | [60,61] |
|  | *Capillaria* sp. | [60] |
|  | *Cristitectus congeri** | [62] |
|  | *Hysterothylacium aduncum* | [60] |
|  | *Neoascarophis* sp. | [60,61] |
|  | *Spinitectus oviflagellis* | [60] |
|  | Nematoda indet. | [60,63] |
|  | **Crustacea (1)** |  |
|  | *Chondracanthodes deflexus* | [60] |
|  |  |  |
| ***Coryphaenoides mexicanus*** | **Digenea (3)** |  |
| Caribbean | *Gonocerca phycidis* | [64] |
|  | *Steringophorus* sp. | [64] |
|  | *Lepocreadiidae* indet. | [64] |
|  |  |  |
| ***Coryphaenoides nasutus*** | **Crustacea (4)** |  |
| North Pacific | *Chelonichondria okamurai** | [46] |
|  | *Clavella okamurai* | [58] |
|  | *Clavella sokodara* | [58] |
|  | *Lophoura ventricul** | [54] |
|  |  |  |
| ***Coryphaenoides profundicolus*** | **Digenea (2)** |  |
| North Atlantic | *Lepidapedon zubchenkoi* | [42,55] |
|  | *Steringophorus thulini* | [47] |
|  |  |  |
| ***Coryphaenoides rupestris*** | **Digenea (13)** |  |
| North Atlantic | *Aporocotyle simplex** | [2] |
|  | *Derogenes varicus* | [2] |
|  | *Dinosoma* sp. | [5] |
|  | *Dolichoenterum* sp. | [65,66] |
|  | *Glomericirrus macrouri* | [2,39,67–69] |
|  | *Gonocerca crassa* | [65,66] |
|  | *Gonocerca kobayashii* | [5] |
|  | *Gonocerca macroformis* | [2] |
|  | *Gonocerca phycidis* | [2,39,66–68] |
|  | *Lecithophyllum botryophorum* | [5] |
|  | *Paraccacladium jamiesoni* | [39,68,70] |
|  | *Parahemiurus merus** | [66] |
|  | *Steringophorus* sp. | [8] |
|  | **Monogenea (1)** |  |
|  | *Macrouridophora macruri* | [2,5,19,39,66,68,71] |
|  | **Cestoda (5)** |  |
|  | *Bothriocephalus* sp.* | [5] |
|  | *Nybelinia* sp. | [39] |
|  | *Philobythos atlanticus* | [2,68] |
|  | Pseudophyllidea indet | [2,66,72] |
|  | *Scolex pleuronectis* | [2,5] |
|  | **Nematoda (6)** |  |
|  | *Anisakis simplex* | [39,73] |
|  | *Anisakis* sp. | [5,66] |
|  | *Contracaecum* sp. | [8] |
|  | *Fellicola longispiculus** | [73,74] |
|  | *Hysterothylacium aduncum* | [2,5,39,72] |
|  | Nematoda indet. | [39,72] |
|  | **Acanthocephala (1)** |  |
|  | *Echinorhynchus gadi* | [66] |
|  | **Crustacea (3)** |  |
|  | *Chondracanthodes radiatus* | [2] |
|  | *Clavella adunca* | [2] |
|  | Crustacea indet. | [5] |
|  |  |  |
| ***Coryphaenoides serrulatus*** | **Digenea (3)** |  |
| South Pacific | *Lepidapedon cascadense* | [36] |
|  | *Lepidapedon oregonense* | [36] |
|  | *Lepidapedon yaquina* | [36] |
|  | **Monogenea (4)** |  |
|  | *Diclidophora tubiformis* | [75] |
|  | *Diclidophora* sp. | [23,76] |
|  | *Polycliphora nezumiae** | [75,76] |
|  | *Polycliphora* sp.* | [23] |
|  | **Crustacea (1)** |  |
|  | *Clavella deminuta** | [23] |
|  |  |  |
| ***Coryphaenoides striaturus*** | **Digenea (1)** |  |
| global | *Botulus microporus** | [77] |
|  | **Cestoda (1)** |  |
|  | *Nybelinia* sp. | [10] |
|  |  |  |
| ***Coryphaenoides subserrulatus*** | **Monogenea (2)** |  |
| South Pacific | *Diclidophora tubiformes* | [75] |
|  | *Diclidophora* sp. | [76] |
|  | **Crustacea (1)** |  |
|  | *Lophoura bipartita** | [54] |
|  |  |  |
| ***Coryphaenoides zaniophorus*** | **Digenea (5)** |  |
| Caribbean | *Gonocerca phycidis* | [64] |
|  | *Lepidapedon mexicanense* | [52] |
|  | *Lepidapedon desotoense* | [52] |
|  | *Lepidapedon zaniophori* | [52] |
|  | *Lepidapedon* sp. | [64] |
|  | **Monogenea (1)** |  |
|  | *Syncoelicotyloides zaniophori** | [78] |

**Supplementary references**

1. Kabata Z. Anew genus and species of trematode parasitic in *Macrurus fabrici* (Sundeval), a deep-sea fish. Proc. Zool. Soc. Lond. Wiley Online Library; 1961.

2. Zubchenko AV. Parasitic fauna of some Macrouridae in the Northwest Atlantic. J. Northwest Atl. Fish. Sci. 1981;2:67–72.

3. Klimpel S, Palm HW, Busch MW, Kellermanns E, Rückert S. Fish parasites in the Arctic deep-sea: Poor diversity in pelagic fish species vs. heavy parasite load in a demersal fish. Deep Sea Res. Part Oceanogr. Res. Pap. 2006;53:1167–1181.

4. Palm HW, Klimpel S. Metazoan fish parasites of *Macrourus berglax* Lacepède, 1801 and other macrourids of the North Atlantic: Invasion of the deep sea from the continental shelf. Deep Sea Res. Part II Top. Stud. Oceanogr. 2008;55:236–242.

5. Zubchenko AV. On the fauna of Macrouridae parasites in North Atlantic. Tr Poljarn Nauchno-Issled Inst Morsk Rybn Choz Okeanogri PINRO. 1975;35:234–238.

6. Bray RA. Digenea in marine fishes from the eastern seaboard of Canada. J. Nat. Hist. 1979;13:399–431.

7. Campbell RA. A new digenetic trematode, *Gibsonia borealis* sp. n. (Lepocreadiidae: Lepidapedinae), parasitic in the rattail *Macrourus berglax* from the Flemish Cap off Newfoundland. J. Helminthol. Soc. Wash. 1992;59:5–8.

8. Houston KA, Haedrich RL. Food habits and intestinal parasites of deep demersal fishes from the upper continental slope east of Newfoundland, northwest Atlantic Ocean. Mar. Biol. 1986;92:563–574.

9. Campbell RA, Correia SJ, Haedrich RL. A new monogenean and cestode from the deep-sea fish, *Macrourus berglax* Lacépède, 1802, from the Flemish Cap off Newfoundland. Proc. Helminthol. Soc. Wash. 1982;49:169–175.

10. Parukhin AM. Helminths of benthic fish of the Southern Ocean. Kiev: Naukowa Dumka; 1989. p. 7–107.

11. Moravec F, Klimpel S, Kara E. *Neoascarophis macrouri* n. sp. (Nematoda: Cystidicolidae) from the stomach of *Macrourus berglax* (Macrouridae) in the eastern Greenland Sea. Syst. Parasitol. 2006;63:229–235.

12. Moravec F, Klimpel S. New data on the morphology of *Spinitectus oviflagellis* Fourment, 1884 (Nematoda: Cystidicolidae) from the pyloric caeca of *Macrourus berglax* (Macrouridae) in the eastern Greenland Sea. Syst. Parasitol. 2007;67:43–50.

13. Ho J-S. Copepod parasites of deep-sea benthic fishes from the western North Atlantic. Parasitology. 1985;90:485–497.

14. Priebe K. Befall des nordatlantischen Grenadierfisches *Macrourus berglax* mit einem Kopepoden der Gattung *Sphyrion*. Arch. Lebensm.-Hyg. 1980.

15. Bakay YI. On infestation of marine redfishes (*Sebastes genus*) of the North Atlantic by the copepod *Sphyrion lumpi* (Krøyer, 1845). Proc. Workshop *Sphyrion Lumpi* Krøyer 1845 Güstrow GDR. 1989. p. 29–36.

16. Gaevskaya AV, Rodjuk GN. Ecological characteristics of the parasitofauna of the macrourid *Macrourus carinatus* Günther in the South Atlantic. Nauchnye Dokl. Vysshei Shkoli Biol. Nauki. 1988;2:21–25.

17. Gaevskaya AV, Rodjuk GN. New and rare Trematoda species from deep-sea fishes of the south west Atlantic. Vestn. Zool. 1988;5:11–15.

18. Mamaev YL, Parukhin AM, Zubtschenko AV. *Diclidophora paracoelorhynchi* and *D. attenuata*, new species of monogeneans from macrourid fishes. Tr. Gelmitologicheskoi Lab. 1979;29:97–102.

19. Rubec LA, Dronen NO. Revision of the genus *Diclidophora* Krøyer, 1838 (Monogenea: Diclidophoridae), with the proposal of *Macrouridophora* ng. Syst. Parasitol. 1994;28:159–185.

20. Gaevskaya AV, Rodjuk GN. A new data on fish trematode fauna of the south-west Atlantic. Parazitologiya. 1983;3:28–32.

21. Zdzitowiecki K, Cielecka D. Digenea of the fish, *Macrourus holotrachys* [Gadiformes, Macrouridae] from the North Scotia Ridge, sub-Antarctic. Acta. Parasitol. 1998.

22. Gaevskaya AVI, Kovaleva AA. Data on the trematodes of fish from the south-western Atlantic. Vestn. Zool. 1978;60–66.

23. Rohde K, Hayward C, Heap M. Aspects of the ecology of metazoan ectoparasites of marine fishes. Int. J. Parasitol. 1995;25:945–970.

24. Gaevskaya AV, Kovaleva AA. Special features of the monogenean fauna of the southwest Atlantic. Investigation of monogeneans in the USSR. 1977. p. 102–104.

25. Mamaev IL, Brashovian PP. Syncoelicotyloides macruri gen. et sp. n.- the first representative of the subfamily Syncoelidotylinae (Microcotylidae, Monogenea) from macruriform fishes. Parazitologiia. 1989;23:532–536.

26. Rohde K, Ho J-S, Smales L, Williams R. Parasites of Antarctic fishes: Monogenea, Copepoda and Acanthocephala. Mar. Freshw. Res. 1998;49:121–125.

27. Rodjuk G. New species of Acanthocephala of the genus *Echinorhynchus* (Echinorhynchidae) from the southwestern Atlantic. Parazitologiya. 1986;20:224–227.

28. Stadler T. Contribucion al conocimiento de los parasitos de la fauna Antarctica. Parte II *Lophoura* Szidati N Sp Parasito *Macrourus holotrachys* Gunther Crustac. Sphyriidae En Pisces Macrouridae Contrib Inst Antarct Argent. 1978;230:1–13.

29. Zdzitowiecki K, Cielecka D. Digenea of fishes of the Weddell Sea. I. Parasites of *Macrourus whitsoni* (Gadiformes, Macrouridae). Acta Parasitol. 1997;42:23–30.

30. Walter T, Palm H, Piepiorka S, Rückert S. Parasites of the Antarctic rattail *Macrourus whitsoni* (Regan, 1913)(Macrouridae, Gadiformes). Polar Biol. 2002;25:633–640.

31. Zdzitowiecki K. Antarctic Digenea, parasites of fishes. Koenigstein: Koeltz Scientific Books; 1997.

32. Rocka A, Zdzitowiecki K. Cestodes in fishes of the Weddell Sea. Acta Parasitol. 1998;43:64–70.

33. Rocka A. The tetraphyllidean cercoids from teleosts occurring in the Weddell Sea (Antarctic). Acta Parasitol. 1999;44:115–118.

34. Zdzitowiecki K. Acanthocephala in fish in the Weddell Sea (Antarctic). Acta Parasitol. 1996;41:199–203.

35. McCauley JE, Pequegnat JE. Two New Species of *Dinosoma* Manter, 1934 (Trematoda: Hemiuridae) from Deep-Water Macrourid Fishes off the Coast of Oregon. J. Parasitol. 1968;54:931–4.

36. McCauley JE. Six Species of *Lepidapedon* Stafford, 1904 (Trematoda: Lepocreadiidae) from Deep-Sea Fishes. J. Parasitol. 1968;54:496–505.

37. Mamaev YL, Lyadov VN. Monogeneans of the subfamily Diclidophoropsinae (Monogenoidea, Diclidophoridae). Tr Biol-Pochv Inst Vladivostok Nov Ser. 1975;26:115–125.

38. Solovjeva G. *Metabronema insulanum* sp.n. (Nematoda, Spirurina) a parasite of deep-water fishes from the Pacific. Mezhdunarodnaya Kniga 39 Dimitrova Ul., 113095 Moscow, Russia; 1991.

39. Campbell RA, Haedrich RL, Munroe TA. Parasitism and ecological relationships among deep-sea benthic fishes. Mar. Biol. 1980;57:301–313.

40. Campbell RA, Munroe TA. New hemiurid trematodes from deep-sea benthic fishes in the western North Atlantic. J. Parasitol. 1977;285–294.

41. Campbell RA, Bray RA. *Lepidapedon* spp. (Digenea: Lepocreadiidae) from deep-sea gadiform fishes of the NW Atlantic Ocean, including four new species. Syst. Parasitol. 1993;24:99–110.

42. Bray RA, Gibson DI. The Lepocreadiidae (Digenea) of fishes from the north-east Atlantic: a review of the genus Lepidapedon Stafford, 1904. Syst. Parasitol. 1995;31:81–132.

43. Bray RA, Clers SA des. Multivariate analyses of metrical features in the Lepidapedon elongatum (Lebour, 1908) species-complex (Digenea, Lepocreadiidae) in deep and shallow water gadiform fishes of the NE Atlantic. Syst. Parasitol. 1992;21:223–232.

44. Bray RA, Gibson DI. The Acanthocolpidae (Digenea) of fishes from the north-east Atlantic: the status of *Neophasis* Stafford, 1904 (Digenea) and a study of North Atlantic forms. Syst. Parasitol. 1991;19:95–117.

45. Palm H. Untersuchungen zur Systematik von Rüsselbandwürmern (Cestoda: Trypanorhyncha) aus atlantischen Fischen. Institut für Meereskunde; 1995.

46. Ho J-S. Chondracanthid copepods (Poecilostomatoida) parasitic on Japanese deep-sea fishes, with a key to the genera of the Chondracanthidae. J. Nat. Hist. 1994;28:505–517.

47. Bray RA. *Steringophorus* Odhner, 1905 (Digenea: Fellodistomidae) in deep-sea fishes from the northeastern Atlantic, with the description of *Steringophorus margolisi* n.sp. Can. J. Fish. Aquat. Sci. 1995;52:71–77.

48. Kritsky DC, Klimpel S. *Cyclocotyloides bergstadi* n. sp. (Monogenoidea: Diclidophoridae: Diclidophoropsinae) from the Gills of Grenadier, *Coryphaenoides brevibarbis* (Teleostei: Macrouridae), in the Northeast Atlantic Ocean. Comp. Parasitol. 2007;74:23–30.

49. Bray RA, Campbell RA. New plagioporines (Digenea: Opecoelidae) from deep-sea fishes of the North Atlantic Ocean. Syst. Parasitol. 1996;33:101–113.

50. Bray RA, Campbell RA. Fellodistomidae and Zoogonidae (Digenea) of deep-sea fishes of the NW Atlantic Ocean. Syst. Parasitol. 1995;31:201–213.

51. Campbell RA. New tetraphyllidean and trypanorhynch cestodes from deep-sea skates in the western North Atlantic. Proc. Helminthol. Soc. Wash. 1977;44:191–193.

52. Blend CK, Dronen NO, Armstrong HW. Six new species of Lepidapedon Stafford, 1904 (Digenea: Lepocreadiidae) from deep-sea macrourid fishes from the Gulf of Mexico and Caribbean Sea, with revised keys to the species of the genus. Syst. Parasitol. 2000;45:29–51.

53. Castro R. parasitic on Chilean fishes in the South Pacific. Acta Parasitol. 2001;46:24–29.

54. Ho J, Kim I-H. *Lophoura* (Copepoda: Sphyriidae) parasitic on the rattails (Pisces: Macrouridae) in the Pacific, with note on *Sphyrion lumpi* from the Sea of Japan. 1989.

55. Bray RA, Gibson DI. The Lepocreadiidae Odhner, 1905 (Digenea) of fishes from the north-east Atlantic: summary paper, with keys and checklists. Syst. Parasitol. 1997;36:223–228.

56. Kuramochi T. Digenean Trematodes of Fishes from Deep-sea Areas off the Pacific Coast of Northern Honshu, Japan. Natl. Mus. Nat. Sci. Monogr. 2009;25–37.

57. Machida M, Kamegai S. Digenean trematodes from deep-sea fishes of Suruga Bay, central Japan. Natl. Sci. Mus. Monogr. 1997;12:19–30.

58. Ho J-S. New species of Clavella (Copepoda: Lernaeopodidae) parasitic on Japanese rattails (Pisces: Macrouridae). 1993.

59. Gibson DI. *Allopodocotyle margolisi* n.sp. (Digenea: Opecoelidae) from the deep-sea fish *Coryphaenoides* (Chalinura) *mediterraneus* in the northeastern Atlantic. Can. J. Fish. Aquat. Sci. 1995;52:90–94.

60. Kellermanns E, Klimpel S, Palm H. Parasite fauna of the Mediterranean grenadier *Coryphaenoides mediterraneus* (Giglioli, 1893) from the Mid-Atlantic Ridge (MAR). Acta Parasitol. 2009;54:158–164.

61. Moravec F, Klimpel S. Two new species of cystidicolid nematodes from the digestive tract of the deep-sea fish *Coryphaenoides mediterraneus* (Giglioli)(Macrouridae) from the Mid-Atlantic Ridge. Syst. Parasitol. 2009;73:37–47.

62. Klimpel S, Busch MW, Kellermanns E, Kleinertz S, Palm H. Metazoan Deep Sea Fish Parasites. Verlag Natur & Wissenschaft; 2009.

63. Mauchline J, Gordon JDM. Feeding and bathymetric distribution of the gadoid and morid fish of the Rockall Trough. J. Mar. Biol. Assoc. U. K. 1984;64:657–665.

64. Blend CK. The digenetic trematodes parasitizing macrourid fishes from the Gulf of Mexico and Caribbean Sea: an examination of their systematics, zoogeography and host-parasite ecology. Texas A&M Univ.; 1996.

65. Szuks H. Zum Befall von *Macrourus rupestris* (Gunnerus) aus dem Bereich von Labrador mit digenen Trematoden. Wiss. Z. Pädagog. Hochsch. Lise. Herrmann. 1975;2:225–231.

66. Szuks H. Verwendbarkeit von Parasiten zur Gruppentrennung beim Grenadierfisch *Macrourus rupestris*. Angew. Parasitol. 1980.

67. Gaevskaya AV. Two new species of trematodes *Gonocerca macrouri* sp. n. and *Hemiurus macrouri* sp. n. from *Macrourus rupestris* L. of the north-east Atlantic. Parazitologiya. 1975;9:457–459.

68. Zubchenko AV. Use of parasitological data in studies of the local grouping of rock Grenadier, *Coryphaenoides rupestris* Gunner. Natl. Oceanogr. Atmospheric Tech. Rep. NMFS. 1985;

69. Gibson DI, Bray RA. Hemiuridae (Digenea) of fishes from the north-east Atlantic. British Museum (Natural History); 1986.

70. Bray RA, Gibson DI. The Accacoeliidae (Digenea) of fishes from the north-east Atlantic. Trustees of the British Museum (Natural History); 1977.

71. Pascoe PL. Monogenean parasites of deep-sea fishes from the Rockall Trough (N.E. Atlantic) including a new species. J. Mar. Biol. Assoc. U. K. 1987;67:603–622.

72. Noble ER, Orias JD, Rodella TD. Parasitic fauna of the deepsea fish, *Macrourus rupestris* (Gunnerus) from Korsfjorden, Norway. Sarsia. 1972;50:47–50.

73. Køie M. Nematode parasites in teleosts from 0 to 1540 m depth off the faroe islands (The North Atlantic). Ophelia. 1993;38:217–243.

74. Petter AJ, Køie M*. Fellicola longispiculus* gen. nov., sp. nov. (Nematoda, Rhoabdochonidae) from the gall bladder of the marine fish *Coryphaenoides rupestris*. Ann. Parasitol. Hum. Comparée. 1993;68:226–228.

75. Rohde K, Williams A. Taxonomy of monogeneans of deep sea fishes in southeastern Australia. Syst. Parasitol. 1987;10:45–71.

76. Rohde K. Gill Monogenea of deepwater and surface fish in southeastern Australia. Hydrobiologia. 1988;160:271–283.

77. Bray RA, others. The bathymetric distribution of the digenean parasites of deep-sea fishes. Folia Parasitol. (Praha). 2004;51:268–274.

78. Rubec LA, Blend CK, Dronen NO. *Syncoelicotyloides zaniophori* n. sp. (Monogenea: Microcotylidae) from the Gills of *Coryphaenoides zaniophorus* (Macrouridae) from the Gulf of Mexico. J. Parasitol. 1995;81:957–960.
